# Implementing relational continuity in general practice—understanding who needs it, when, to what extent, how and why: a realist review protocol

**DOI:** 10.1136/bmjopen-2025-104081

**Published:** 2025-09-09

**Authors:** Victoria Tzortziou Brown, Sophie Park, Kamal Ram Mahtani, Stephanie Taylor, Emily C Owen-Boukra, Jonathan Taylor, Owen Richards, Sultana Begum, Geoff Wong

**Affiliations:** 1 Wolfson Institute of Population Health, Queen Mary University of London, London, England, UK; 2 Nuffield Department of Primary Care Health Sciences, University of Oxford, Oxford, England, UK; 3 Public co-applicant, London, UK

**Keywords:** Primary Care, Patient-Centered Care, Health policy, Systematic Review

## Abstract

**Abstract:**

**Introduction:**

Relational continuity of care (RCC) refers to the sustained therapeutic relationship between a patient and a clinician, which fosters trust, enhances communication and facilitates the accumulation of knowledge about the patient. RCC is associated with enhanced patient outcomes, reduced hospital admissions, lower mortality rates, decreased healthcare costs and improved patient experience. Despite these benefits, reorganisations within the NHS and workforce challenges have led to an increased reliance on multidisciplinary and part-time working, resulting in fragmented care and a decline in RCC. Our study aims to explore who needs RCC, under what circumstances, to what extent and why, with the goal of informing optimal implementation strategies.

**Methods and analysis:**

We will conduct a realist review to develop an evidence-based programme theory explaining the mechanisms underlying RCC, the populations that benefit most, the contextual factors influencing RCC and effective care models. Following Pawson’s five iterative stages, we will: (1) Locate existing theories, (2) Search for relevant evidence, (3) Select appropriate articles, (4) Extract and organise data and (5) Synthesise findings to draw conclusions. A stakeholder advisory group, comprising policymakers, healthcare professionals, public contributors and patients, will be engaged throughout the process. We will adhere to Realist And Meta-narrative Evidence Synthesis: Evolving Standards (RAMESES) for realist reviews to ensure methodological rigor.

**Dissemination and ethics:**

Our findings will inform practical, evidence-based recommendations for optimising RCC within general practice. Outputs will include peer-reviewed publications, conference presentations, plain English summaries, social media infographics, a short video and end-of-study events. Collaborations with stakeholders and public involvement will ensure both accessibility and impact. Ethical approval is not required for this review.

**STRENGTHS AND LIMITATIONS OF THIS STUDY**

- The use of a realist review is a methodological strength, as it allows for the systematic and theory-informed synthesis of diverse evidence relevant to relational continuity in general practice (GP) settings, accommodating the complexity and contextual variation inherent in this area.
- An initial programme theory has already been developed through reference to formal theory and feedback provided during patient and public involvement (PPI) and engagement activities.
- A stakeholder group will be consulted throughout the review to ensure that the review focuses on key policy aspects, enhancing their impact and relevance.
- Public co-applicants have co-authored the review protocol and PPI members will be involved throughout the design, analysis and reporting stages of the project, contributing diverse perspectives, including those from underserved populations.
- Our context-mechanism-outcome configurations and refined programme theory may be constrained by the availability, quality and depth of the existing literature in this field.

## Introduction

 Relational continuity refers to the sustained therapeutic relationship between a patient and healthcare professionals, which fosters trust, communication and clinical responsibility.^1^ Relational continuity of care (RCC) in general practice is linked to improved patient outcomes, reduced hospital admissions, lower mortality rates, decreased healthcare costs and an enhanced patient experience.^2^,^4^ Despite these benefits, there is little consensus on how to deliver RCC effectively and for whom it should be prioritised.

NHS reorganisations, workforce shortages and the expansion of multidisciplinary teams have contributed to a fragmented healthcare system, resulting in reduced RCC. Policies that prioritise timely access over continuity have further eroded RCC, with only 16% of patients in 2023 consistently able to see their preferred GP.^5^ Calls for incentivising RCC through contractual changes have emerged,^6^ but it remains unclear how to achieve this within the current general practice framework.

Although RCC has demonstrated significant benefits, its impact varies across patient groups.^7^ For example, for patients with chronic conditions, such as diabetes or hypertension, greater continuity of care has been associated with reduced hospitalisation and mortality rates.^8^ However, it is unclear whether these benefits are similar for patients with other long-term conditions. Additionally, patients with cancer receiving RCC are more likely to be able to understand and manage their condition, experience greater feelings of control and have higher overall well-being.^9^ But there is a lack of clarity regarding the benefits of maintaining such RCC after cancer remission and recovery. A recent study showed that the productivity benefit of continuity of care seems to be greater for older patients, those with multiple chronic conditions and individuals with mental health conditions.^10^ However, the evidence on the differential benefits of continuity across various patient groups has not yet been synthesised.^11^

RCC may also enhance patient safety by reducing diagnostic delays, particularly for complex cases.^6^ However, while RCC fosters trust and holistic care, there are potential downsides, including diagnostic bias, patient collusion and increased GP workload, which may contribute to clinician burnout.^12^,^14^

Evidence suggests that RCC is less accessible to patients from non-white ethnic backgrounds and socioeconomically deprived communities, despite their higher preference for continuity.^15 16^ Older individuals also experience reduced RCC, highlighting systemic inequalities in access.^17^ The implementation of RCC can be more challenging in practices situated in areas of high deprivation. This is because general practice in deprived areas is underfunded and under-doctored relative to need.^18^ Evidence suggests a negative association between larger practice sizes and continuity,^19^ with single-handed practices having higher patient-reported access, continuity and overall satisfaction, while higher levels of practice deprivation are associated with lower RCC.^20^

There is an urgent need to identify effective care models that balance RCC with the realities of modern general practice. Understanding which patients benefit most, under what circumstances, and how RCC can be implemented in a multidisciplinary setting is crucial for informing policy and practice. By addressing these gaps, RCC can be optimised to improve patient outcomes, reduce health inequalities and support the sustainability of primary care.

## Methods

### Research question

Implementing relational continuity in general practice—who needs it, when, to what extent, how and why?

### Aims

We aim to understand:

1. The underlying mechanisms through which RCC and its benefits are achieved.
2. The populations of patients and clinicians for whom RCC should be prioritised.
3. The contextual conditions that constrain or facilitate RCC.
4. The care models and interventions that are likely to lead to optimal implementation of RCC.

### Objectives

To address our research’s aims, we will:

- Conduct a realist review of the UK and relevant international literature of RCC in general practice to develop an evidence-based programme theory of its optimal implementation.
- Use the knowledge contained within the programme theory to develop relevant outputs for knowledge users and recommendations for best practice.

### Approach

A realist review methodology will be used in this study. Realist reviews are particularly suited for the study of phenomena, such as RCC, which are inherently complex and multifaceted.^21^ Realist reviews aim to unpack the dynamic interactions between contexts, mechanisms and outcomes of interest to explain causation within interventions or phenomena.^22^

### Engaging with key policy, practice and research stakeholders and with patients and public representatives

Stakeholder, patient and public engagement is essential in this project. It will help us define the review’s focus and provide additional insights into the challenges and opportunities of optimally implementing RCC in general practice. It will also enable additional material (local knowledge and unpublished materials) to be identified. We will discuss emerging findings, ‘sense-check’ and further refine recommendations. This will help ensure the appropriateness of our outputs and the effectiveness of our dissemination strategy. Further, our engagement with stakeholders will leverage support for the ultimate implementation of recommendations and help to identify barriers to implementing positive changes.

We have two patient and public involvement (PPI) co-applicants who have already contributed to this protocol. The PPI group participants have been purposefully selected to ensure diversity in terms of lived experience and representation of populations that have been underserved by research. The stakeholder group includes individuals who are representatives of providers, commissioners, public policy-makers and academics.

Key steps in the review will include the following over a period of 18 months:

### Step 1: locate existing theories

**Figure 1:**
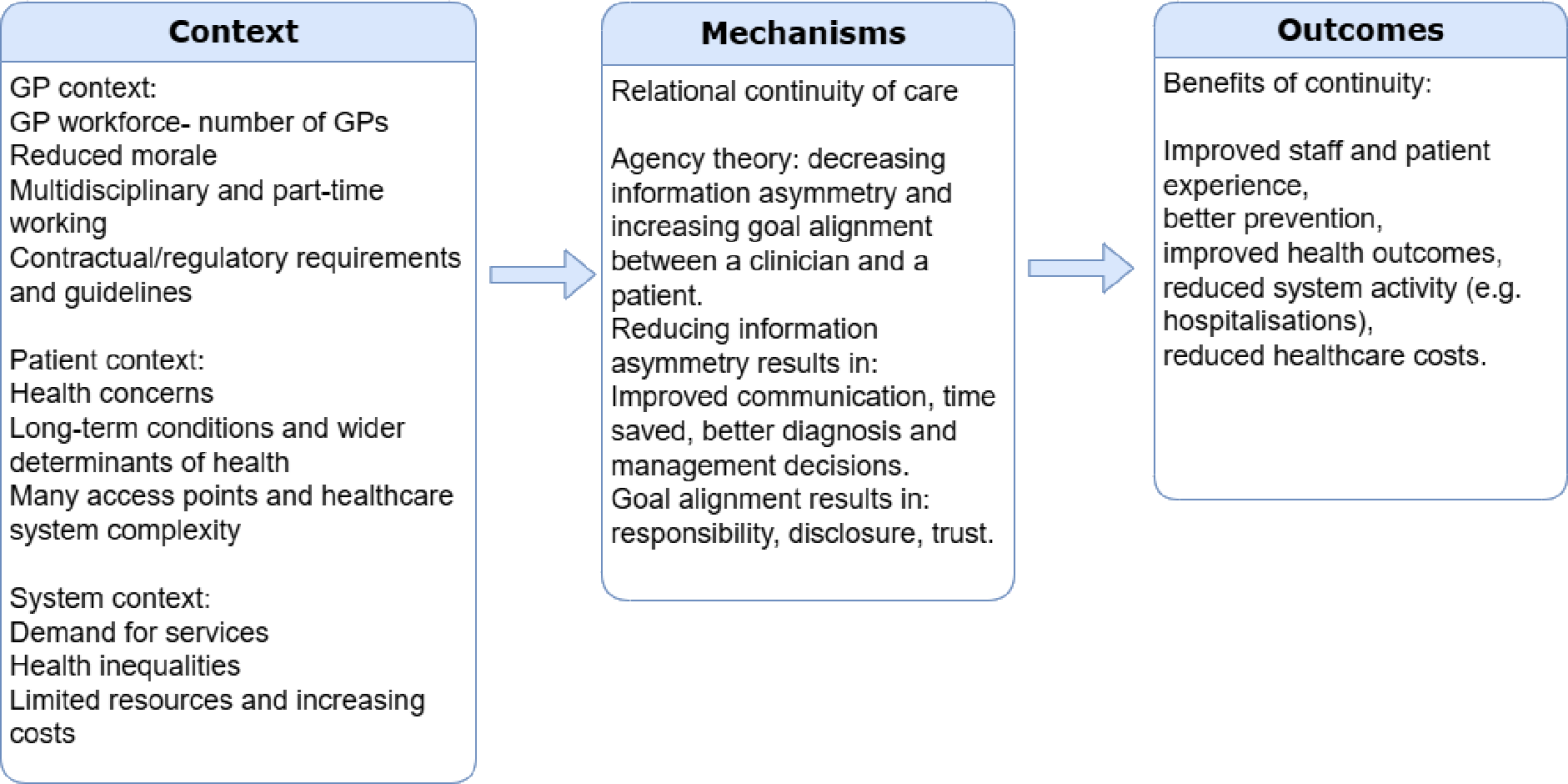
Initial programme theory with unconfigured possible contexts, mechanisms and outcomes of interest.

The goal of this step is to identify underlying programme theories for RCC in general practice—that is, theories that explain how RCC works, in what ways, and with what consequences. Through our early discussions and review of the literature so far, we have begun to identify the possible contexts, mechanisms and outcomes that underlie RCC (see figure 1). We have based this initial theory on agency theory, which proposes that the value of continuity lies in reducing agency loss by decreasing information asymmetry and increasing goal alignment between a clinician and a patient.^23^ Continuity can help reduce information asymmetry about the patient’s health condition, needs, values and preferences, ensuring that the clinician can tailor clinical care to the patient’s specific needs. Continuity can also assist with goal alignment, as it can be viewed as a way of forming a bond that may counteract incentives to diminish the clinician’s intention to act as the perfect agent.^23^ Recent work by Sidaway-Lee *et al*^24^ attempted to summarise various factors that link continuity to patient outcomes, and we have integrated some of these factors into our initial programme theory.

This initial programme theory will be further developed in this first step of the review. We will employ a systematic method, such as the APEASE criteria,^25^ to help identify the most important mechanisms within the programme theories that trigger outcomes during our meetings with stakeholders and PPI groups.

The research team will then conduct a preliminary exploratory literature search to establish the potential scale of the evidence base, the parameters of the review and the scope. The search will apply the following search terms: continuity AND (relational OR interpersonal) AND general practice AND (interventions OR implementation OR evaluation) to MEDLINE (Ovid), Web of Science (Social Science Citation Index), Google Scholar and the NHS Knowledge and Library Hub. Findings will inform the development of an initial programme theory or theories, which will be shared with the advisory groups for feedback, modification and refinement.

### Step 2: searching for evidence

The team will develop a more comprehensive search strategy, based on outputs from the previous stage. The search will be informed by the initial programme theory and by the context-intervention-mechanism-outcome framework for searching:

- Context—General practice within the UK and internationally.
- Intervention—Any intervention to facilitate RCC, including targeted (if applicable) patient populations, and any reports of experiences with such interventions by patients, their carers and GP staff.
- Mechanism—to be informed by the preliminary literature search and discussion with the stakeholder group.
- Outcome—Improved RCC, with impacts on patient and staff experience, patient outcomes, GP workforce, service processes and costs.

Secondary evidence will be identified from databases (eg, MEDLINE (OVID), EMBASE (OVID)), health policy think tanks (eg, Nuffield Trust, The King’s Fund, The Health Foundation), NHS repositories (eg, NHS Knowledge and Library Hub) and grey literature. A more detailed account of relevant databases, registers and websites is presented in online supplemental file 1. Where needed, additional searches will be developed iteratively and purposively, to enable the continuing identification of literature to inform development and testing (confirming, refuting or refining) of the programme theory.

#### Additional searching

An important process in realist reviews is searching for additional data to inform programme theory development. In other words, more searches will be undertaken if we find that we require more data to develop and test certain sub-sections of the programme theory.

### Step 3: article selection

The results from the search in Step 2 will be de-duplicated. There will be two stages of screening: (1) Title and abstract screening and (2) Full text screening.

Records will be screened by one reviewer, and at each stage, a 10% random sample will be independently screened to check for systematic errors. Disagreements will be resolved via discussion and the involvement of a third team member if necessary. Full-text documents will be assessed for relevance, richness and rigour.

Relevance will consider whether an article aligns with the topic and supports theory development. Richness will reflect the depth of data informing Context, Mechanisms and/or Outcomes (CMOs) within the included documents.

Rigour will follow Wong’s approach, evaluating both data sources (where needed) and the plausibility and coherence of the theoretical explanations developed—that is, the programme theories. We will assess the plausibility and coherence of the programme theories using three criteria: consilience (whether they can explain the widest range of data), simplicity (avoiding unnecessary complexity) and analogy (alignment with existing knowledge).^26^

Where necessary, individual documents will undergo quality appraisal, particularly if they provide key evidence for a programme theory. All appraisal decisions will be transparently documented and used to report the confidence in the strength of any knowledge claims made.

### Step 4: extracting and organising data

Data from all relevant full-text documents will be extracted using a suitably designed and piloted systematic data collection process. Key characteristics of each included document will be extracted into an Excel spreadsheet, and the full text of the documents will be uploaded to NVivo qualitative data analysis software, allowing relevant data to be organised and coded. Coding will involve extracting relevant sections of text from included documents according to how this data can contribute to refining programme theory. This coding will be inductive (codes created to categorise data reported in included studies), deductive (codes created in advance of data extraction and analysis as informed by the initial programme theory) and retroductive (codes created based on inferences as to what may be functioning as mechanisms).

### Step 5: evidence synthesis and drawing conclusions

Data analysis will be conducted in NVivo, and coding will use inductive, deductive and retroductive approaches to support the interpretation and development of context-mechanism-outcome-configurations (CMOCs) and the programme theory or theories. We will use a series of questions to guide our realist analysis of the data from the included articles. In brief, to operationalise the realist logic of analysis, we will ask the following questions:^27^

- Interpretation of meaning: do the documents provide data that can be interpreted as functioning as context, mechanism or outcome?
- Interpretations and judgements about CMOCs: what is the CMOC for the data that has been interpreted as functioning as context, mechanism or outcome?
- Interpretations and judgements about programme theory: how does this CMO relate to the initial programme theory?

Data synthesis will involve team discussions to assess the integrity of programme theories, evaluate supporting evidence, compare theories across settings and align findings with practitioner and patient experiences. Our goal is to develop a detailed, context-specific programme theory explaining how to implement RCC in NHS general practice.

Stakeholders and PPI advisory group members will review and refine the theory through meetings and workshops, offering content expertise and real-world validation. Their feedback will ensure alignment with practical experiences, leading to a well-informed, consensus-driven final programme theory.

We will use data from our review, as well as feedback and advice received from workshops with our stakeholders and PPI groups, to inform the development of practical recommendations on the optimal implementation of RCC and identify evidence gaps and future research directions.

## Ethics and dissemination

### Dissemination

Our findings will be used to develop evidence-based, user-friendly, practical recommendations for the prioritisation and optimal implementation of RCC. We will collaborate with our stakeholder group, PPI members, and an artist/infographics expert to jointly develop outputs to reach different audiences, including: GP practices, policy makers, commissioners, academics, patients and the public. The study findings will be shared via conference abstracts and presentations, at least one peer-reviewed publication in a high-impact journal, a plain English summary, two end-of-study events, a series of social media infographics and a short video.

### Ethics

Ethical review is not required for this review as only secondary data sources will be used (Queen Mary Ethics Committee QME24.1030).

## Supplementary material

10.1136/bmjopen-2025-104081 online supplemental file 1
