# Supplementary material for: Implementing relational continuity in general practice—understanding who needs it, when, to what extent, how and why: a realist review protocol
Source: BMJ Open. 2025 Sep 9;15(9):e104081. doi: 10.1136/bmjopen-2025-104081 (PMC12421597; doi:10.1136/bmjopen-2025-104081)
Supplement: online supplemental file 1 [file bmjopen-15-9-s001.docx]

**Supplementary File 1**

**Preliminary search strategy**

This document outlines the initial approach to the search strategy for the realist review on continuity of care. It includes a preliminary list of databases, websites, and grey literature sources to be searched. This strategy is subject to refinement through ongoing consultation with our stakeholders and Public and Patient Involvement (PPI) groups to ensure it is comprehensive and contextually appropriate.

At present, the search will focus on the following sources:

**Databases**:

We will search the following electronic databases from the beginning of the databases to present: - .

- MEDLINE (Ovid),
- Embase,
- CINAHLPlus,
- PsycINFO,
- Scopus,
- AMED (OVID)
- Cochrane
- Academic Search Complete (EBSCO)
- Web of Science (SCI, SSCI, CPCI-S, CPCI-SSH)
- Applied Social Sciences Index and Abstracts (ASSIA) (ProQuest),
- Epistemonikos.

**Trial Registers**:

- ClinicalTrials.gov,
- WHO ICTRP.

**Grey Literature and Websites**:

- National Institute for Health and Care Excellence (NICE) – <https://www.nice.org.uk>
- National Health Service (NHS England and NHS Digital) – https://www.england.nhs.uk, <https://digital.nhs.uk>
- Royal College of General Practitioners <https://www.rcgp.org.uk/>
- The King’s Fund – <https://www.kingsfund.org.uk>
- Health Foundation (UK) – <https://www.health.org.uk>
- Nuffield Trust – <https://www.nuffieldtrust.org.uk>
- World Health Organization (WHO) – <https://www.who.int>
- Agency for Healthcare Research and Quality (AHRQ, USA) – <https://www.ahrq.gov>
- Canadian Agency for Drugs and Technologies in Health (CADTH) – <https://www.cadth.ca>
- Australian Commission on Safety and Quality in Health Care – <https://www.safetyandquality.gov.au>
- International Foundation for Integrated Care (IFIC) – <https://integratedcarefoundation.org>
- Patient Information Forum (PIF) – <https://www.pifonline.org.uk>
- Healthtalk.org (patient experiences) – <https://www.healthtalk.org>
- OpenGrey (for European grey literature) – <http://www.opengrey.eu>
- Gov.uk – Publications and guidance from the UK Department of Health and Social Care – <https://www.gov.uk/government/organisations/department-of-health-and-social-care>
- Health Management Information Consortium (HMIC) (OVID)
- ETHOS (British Library Electronic Thesis Online) <https://libguides.ials.sas.ac.uk/az/ethos-electronic-theses-online-system>
- Networked Digital Library of Theses and Dissertations (NDLTD) <https://ndltd.org/>
- NHS Knowledge and Library Hub <https://library.nhs.uk/knowledgehub/>

Additional sources may be added based on suggestions from stakeholders and as snowballing identifies further relevant organisations and reports. The anticipated start date for the searches will be June 1, 2025.
